# Listeria monocytogenes in Stone Fruits Linked to a Multistate Outbreak: Enumeration of Cells and Whole-Genome Sequencing

**DOI:** 10.1128/AEM.01486-16

**Published:** 2016-11-21

**Authors:** Yi Chen, Laurel S. Burall, Yan Luo, Ruth Timme, David Melka, Tim Muruvanda, Justin Payne, Charles Wang, George Kastanis, Anna Maounounen-Laasri, Antonio J. De Jesus, Phillip E. Curry, Robert Stones, Okumu K'Aluoch, Eileen Liu, Monique Salter, Thomas S. Hammack, Peter S. Evans, Mickey Parish, Marc W. Allard, Atin Datta, Errol A. Strain, Eric W. Brown

**Affiliations:** a Center for Food Safety and Applied Nutrition, Food and Drug Administration, College Park, Maryland, USA; b Center for Food Safety and Applied Nutrition, Food and Drug Administration, Laurel, Maryland, USA; c Newcastle University, Newcastle upon Tyne, United Kingdom; d Office of Regulatory Affairs, Food and Drug Administration, Alameda, California, USA; Pennsylvania State University

## Abstract

In 2014, the identification of stone fruits contaminated with Listeria monocytogenes led to the subsequent identification of a multistate outbreak. Simultaneous detection and enumeration of L. monocytogenes were performed on 105 fruits, each weighing 127 to 145 g, collected from 7 contaminated lots. The results showed that 53.3% of the fruits yielded L. monocytogenes (lower limit of detection, 5 CFU/fruit), and the levels ranged from 5 to 2,850 CFU/fruit, with a geometric mean of 11.3 CFU/fruit (0.1 CFU/g of fruit). Two serotypes, IVb-v1 and 1/2b, were identified by a combination of PCR- and antiserum-based serotyping among isolates from fruits and their packing environment; certain fruits contained a mixture of both serotypes. Single nucleotide polymorphism (SNP)-based whole-genome sequencing (WGS) analysis clustered isolates from two case-patients with the serotype IVb-v1 isolates and distinguished outbreak-associated isolates from pulsed-field gel electrophoresis (PFGE)-matched, but epidemiologically unrelated, clinical isolates. The outbreak-associated isolates differed by up to 42 SNPs. All but one serotype 1/2b isolate formed another WGS cluster and differed by up to 17 SNPs. Fully closed genomes of isolates from the stone fruits were used as references to maximize the resolution and to increase our confidence in prophage analysis. Putative prophages were conserved among isolates of each WGS cluster. All serotype IVb-v1 isolates belonged to singleton sequence type 382 (ST382); all but one serotype 1/2b isolate belonged to clonal complex 5.

**IMPORTANCE** WGS proved to be an excellent tool to assist in the epidemiologic investigation of listeriosis outbreaks. The comparison at the genome level contributed to our understanding of the genetic diversity and variations among isolates involved in an outbreak or isolates associated with food and environmental samples from one facility. Fully closed genomes increased our confidence in the identification and comparison of accessory genomes. The diversity among the outbreak-associated isolates and the inclusion of PFGE-matched, but epidemiologically unrelated, isolates demonstrate the high resolution of WGS. The prevalence and enumeration data could contribute to our further understanding of the risk associated with Listeria monocytogenes contamination, especially among high-risk populations.

## INTRODUCTION

Listeria monocytogenes has been associated with foodborne outbreaks linked to contaminated ice cream (1), meat (2), caramel apples (3), and cheese (4). Recently, whole-genome sequencing has been employed to assist in listeriosis outbreak investigations (5–9). To do this, bioinformatics tools have been developed to target different genomic variations of L. monocytogenes (single nucleotide polymorphisms, allelic differences, k-mer, etc.) in either the core or whole genome (10–13). In the United States, nationwide real-time whole-genome sequencing (WGS) was implemented using the GenomeTrakr and PulseNet network to enhance listeriosis outbreak detection and investigation (14). In several outbreak investigations, the U.S. Centers for Disease Control and Prevention (CDC) had employed a whole-genome multilocus sequence typing (wgMLST) tool that targets the allelic differences in genome-wide coding regions (14), and the U.S. Food and Drug Administration (FDA) had employed a reference-based Center for Food Safety and Applied Nutrition (CFSAN) SNP Pipeline that identifies single nucleotide polymorphisms (SNPs) in the entire genome, including core genes, accessory genes, and intergenic regions (8, 11, 15).

In July 2014, certain lots of fresh stone fruits, including whole peaches, nectarines, plums, and pluots, were recalled by a packing company (company A) due to contamination with L. monocytogenes (16). Subsequently, two clinical cases were linked to these recalled fruits, which were the first reported human listeriosis cases caused by the consumption of contaminated stone fruit (14, 16). Initially, pulsed-field gel electrophoresis (PFGE) comparisons were performed on nationwide clinical cases reported in PulseNet, with isolation dates between 1 May and 31 August 2014, indicating possible exposure to the recalled fruits. This identified isolates from 4 patients in 4 states (Illinois, South Carolina, Minnesota, and Massachusetts) having PFGE profiles indistinguishable from those of isolates collected from the stone fruits and their packing environment (16). Subsequent epidemiological investigation and WGS analysis suggested that only patients in Minnesota and Massachusetts were likely linked to the contaminated stone fruits (14, 16). The wgMLST phylogeny was reported on isolates from stone fruits and their packing environment obtained in the present study and clinical isolates obtained during the epidemiological investigation (14).

Enumerating L. monocytogenes in food products linked to human listeriosis can provide a data set for our further understanding of the risk associated with L. monocytogenes contamination. Therefore, when isolating L. monocytogenes from fruits in contaminated lots, we performed simultaneous detection and enumeration. To explore whether WGS analyses of outbreak-associated isolates using different tools could lead to the same conclusions as wgMLST, an SNP-based WGS method was also performed on isolates from stone fruits, their packing environment, and patients during the outbreak investigation. This study describes the enumeration and WGS of L. monocytogenes associated with this outbreak.

## MATERIALS AND METHODS

### Fruits.

We randomly collected 105 unwounded stone fruits from seven lots (15 fruits/lot) available at the company's facility during the time of sampling. These lots were on the company's recall list, but the 105 fruits were stored in the packing facility and had never been distributed to commerce. These fruits were physiologically mature, but not fully ripe, at the time of analysis. The seven lots of fruits were composed of organic yellow nectarines, two cultivars of conventional white nectarines, conventional yellow nectarines, organic white peaches, conventional white peaches, and organic yellow peaches. Each fruit weighed between 127 and 145 g, with an average of 132 g.

### Enumeration.

Preliminary analysis did not recover L. monocytogenes from the fruit pulp; therefore, we performed rinsing and direct plating enumeration of L. monocytogenes on the surface of each individual fruit. Briefly, each fruit was submerged in a Whirl-Pak bag (Nasco, Inc., Fort Atkinson, WI) with 80 ml of Butterfield's phosphate buffer (BPB), and the sealed bags were subsequently hand-massaged for 1 min. These bagged fruits were then put in a shaking incubator (Innova 44; Eppendorf, Inc., Hauppauge, NY) at 250 rpm for 5 min at room temperature. The resulting BPB rinsate was centrifuged for 10 min at 3,500 × *g*, and cell pellets were then resuspended in 1 ml of BPB. One hundred microliters of suspended cells was plated onto each of the two ALOA (Agar Listeria according to Ottavani and Agosti) plates (catalog no. AEB520080; bioMérieux, Inc., St. Louis, MO) and two RAPID'L. mono agar plates (catalog no. 3563694; Bio-Rad Laboratories, Inc., Hercules, CA), followed by incubation for up to 48 h at 37°C. A subset of typical colonies were confirmed using the real-time PCR scheme as described in the L. monocytogenes chapter of the FDA's Bacteriological Analytical Manual (17). This enumeration scheme had a lower limit of detection (LOD) of 5 CFU/fruit, and the rinsing generated an average of 75% recovery rate for peaches and 85% recovery rate for nectarines (our unpublished data).

### Statistical analysis.

In order to perform statistics, we assumed the concentration of L. monocytogenes to be half of the LOD (2.5 CFU/fruit) for the fruit that did not yield L. monocytogenes. Medians, geometric means, and arithmetic means were calculated for each lot. Comparisons of medians among different lots were performed using a Mann-Whitney test with Holm-Bonferroni correction (18, 19). Log-transformed values of geometric means were compared with one-way analysis of variance (ANOVA) (20).

### Serotyping and PFGE.

We performed serotyping on the following isolates: one isolate from each of the 56 fruits that yielded L. monocytogenes, and an additional isolate from 38 out of these 56 fruits, as well as 2 fruit isolates and 17 environmental isolates obtained from company A. Serotyping was performed using either a combination of antiserum agglutination and multiplex PCR (21) before WGS was obtained or the *in silico* serotyping tool in the Pasteur MLST L. monocytogenes database (http://bigsdb.web.pasteur.fr/listeria/listeria.html) after WGS was obtained. We performed PFGE on a subset of isolates representing all fruit cultivars, lots, and environmental samples using the standard PulseNet protocol (22). We obtained the PFGE profiles from PulseNet on the 4 clinical isolates identified during the initial epidemiological investigation.

### WGS.

We performed WGS on a subset of isolates representing all fruit cultivars, lots, and environment samples.

DNA was isolated from pure cultures using the Qiagen DNeasy blood and tissue kit (catalog no. 69582; Qiagen, Inc., Valencia, CA). Sequencing libraries were prepared using the Nextera XT sample preparation kit (catalog no. FC-131-1024; Illumina, Inc.), and WGS was performed using a MiSeq (Illumina, Inc., San Diego, CA) with the version 2 kit (2 × 250 bp), according to the manufacturer's instructions (23).

A serotype IVb-v1 isolate (CFSAN023463) and a serotype 1/2b isolate (CFSAN023459), both collected from fruits, were selected to be fully sequenced using PacBio RSII (Pacific Biosciences, Menlo Park, CA, USA), as previously described (24, 25). Briefly, a single 10-kb library was prepared and sequenced using C2 chemistry on 8 single-molecule real-time (SMRT) cells with a 90-min collection protocol on the PacBio RS. These 10-kb continuous-long-read data were then *de novo* assembled using the PacBio Hierarchical Genome Assembly Process (HGAP2.0)/Quiver software package, followed by Minimus 2 to yield a single chromosomal contig and a plasmid contig. Raw reads were subsequently mapped to the contigs using Quiver for error correction. These two complete genomes were annotated with the National Center for Biotechnology Information (NCBI) Prokaryotic Genome Annotation Pipeline (http://www.ncbi.nlm.nih.gov/genomes/static/Pipeline.html). During sequencing, epigenetic modifications at each nucleotide position were measured as kinetic variations (KV) in the nucleotide incorporation rates, and methylase activities were deduced from the KV data (26, 27). The methylomes of these two complete genomes were analyzed and deposited in REBASE (http://tools.neb.com/genomes/view.php?list=0&view_id=35417 and http://tools.neb.com/genomes/view.php?view_id=36969).

### Reference-based SNP analyses.

WGS analyses were performed using the CFSAN SNP pipeline 0.6.1 (8, 11). Briefly, raw reads from each serotype IVb-v1 isolate were mapped to the complete genome of CFSAN023463, and raw reads from each serotype 1/2b isolate were mapped to the complete genome of CFSAN023459, using default settings within Bowtie 2 version 2.2.2 (28). For each SNP analysis, the resulting BAM file was sorted using SAMtools version 1.3.1 (29), and a pileup file for each isolate was produced. These files were then processed using VarScan2 version 2.3.9 (30) to identify high-quality variant sites, using the mpileup2snp option. An in-house Python script was used to parse the .vcf files and construct an initial SNP matrix. We then removed high-density variant sites (≥3 within 1,000 bp in the reference), including 5 among outbreak isolates, 8 between the South Carolina isolate and the outbreak cluster, and 3 among serotype 1/2b isolates. Additional information about these procedures, e.g., codes and instructions, is available at https://github.com/CFSAN-Biostatistics/snp-pipeline. The Genetic Algorithm for Rapid Likelihood Inference (GARLI) (31) was used to infer phylogenies based on each of the SNP matrices. WGS analyses were performed separately on (i) serotype IVb-v1 isolates, including the two clinical isolates not associated with the outbreak; and (ii) serotype 1/2b isolates, with and without an outgroup (CFSAN010068 [NCBI SRA ID: SRR1181548]) of multilocus sequence typing (MLST) sequence type 5 (ST5) from a 2013 U.S. Hispanic-style cheese outbreak (32).

The draft genomes were *de novo* assembled using the CLC Genomics Workbench 8.0.3 (Qiagen, Waltham, MA), and genes containing the SNPs were extracted by BLAST, translated to protein sequences, and then aligned using the global alignment program MUSCLE (33), with default settings. This enabled the identification of SNPs as being synonymous or nonsynonymous and of the amino acid changes for nonsynonymous SNPs. We searched against NCBI, the protein families database (Pfam) (34), and Kyoto Encyclopedia of Genes and Genomes (KEGG) (35) to further identify the putative functions of the genes containing the SNPs.

### Prophage, clonal group, and *inlA* analyses.

We identified putative prophages from the complete genomes of CFSAN023463 and CFSAN023459 using PHAST (36). We then used BLAST to align these putative prophages with the draft genomes and determined the percentage of query coverage (percentage of the query sequence that overlaps the subject sequence) and sequence identity. *In silico* MLST analysis was performed using the CLC Genomics Workbench 8.0.3 (Qiagen, Waltham, MA) and tools in the Pasteur MLST L. monocytogenes database (http://bigsdb.pasteur.fr/listeria/listeria.html). The alignment and mapping tools from the Qiagen CLC Genomics Workbench were used to extract *inlA* sequences to determine whether there were premature stop codons in *inlA*.

### Accession number(s).

The WGS sequences were deposited under their GenBank accession numbers for complete genomes and Sequence Read Archive (SRA) identifiers for draft genomes. We obtained the WGS data from GenomeTrakr on the 4 clinical isolates previously submitted by the CDC, seen in Table 2.

## RESULTS

### Enumeration.

**FIG 1:**
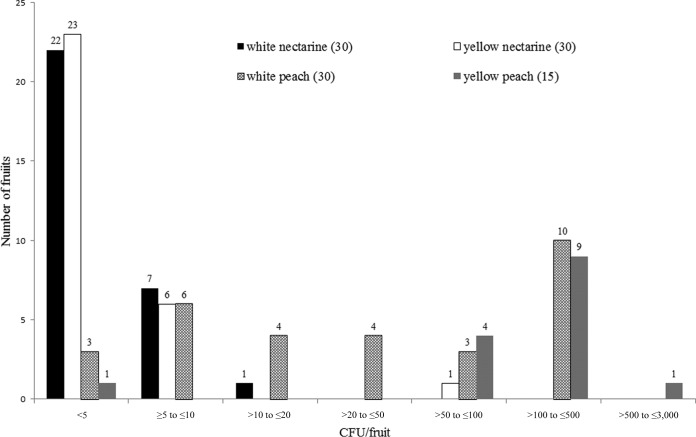
Number of fruits containing L. monocytogenes at various levels. Each fruit weighed 127 to 145 g. The numbers are listed on top of each bar. Different cultivars of the same variety are combined for this analysis. The total number of fruits of each variety is listed in parentheses following the variety name.

Our enumeration scheme (LOD, 5 CFU/fruit) yielded L. monocytogenes on 53.3% (56/105) of all tested fruits (Table 1). Specifically, 25% of the nectarines (15/60) and 91.1% of the peaches (41/45) yielded L. monocytogenes. The geometric means were 2.95, 3.97, 3.16, and 3.79 CFU/fruit for the 4 lots of nectarines, and 14.45, 73.37, and 156.6 CFU/fruit for the 3 lots of peaches. Overall, peaches had higher levels of L. monocytogenes than nectarines (Table 1). Out of the 60 nectarines, 58 and 59 fruits contained L. monocytogenes at ≤10 and ≤20 CFU/fruit, respectively, with the remaining fruit containing L. monocytogenes at 85 CFU/fruit (Fig. 1). Out of the 45 peaches, 44 fruits contained L. monocytogenes at ≤500 CFU/fruit, and the remaining fruit contained L. monocytogenes at 2,850 CFU/fruit (Fig. 1).

**TABLE 1:** Statistical analysis of the enumeration results from seven lots of stone fruits

Stone fruit	CFU/fruit^*a*^					No. of fruits		
	Minimum	Median	Arithmetic mean	Geometric mean	Maximum	Total	Yielding IVb-v1	Yielding 1/2b
White nectarine (cv. 1)	2.5^*b*^	2.5 A	3.5	2.95 A	15	15	2	0
White nectarine (cv. 2)	2.5	2.5 A	4.8	3.97 A	10	15	3^*c*^	4^*c*^
Yellow nectarine	2.5	2.5 A	8.0	3.16 A	85	15	1^*c*^	1^*c*^
Yellow nectarine, organic	2.5	2.5 A	4.5	3.79 A	10	15	4^*c*^	3^*c*^
White peach	2.5	10.0 B	42.3	14.45 B	325	15	13^*c*^	1^*c*^
White peach, organic	2.5	110 C	135.8	73.37 C	495	15	10	4
Yellow peach, organic	2.5	190 C	362.5	156.6 C	2,850	15	13^*c*^	2^*c*^
Total	2.5	5.0	80.2	11.28	2,850	105	46	15

a Each fruit weighed 127 to 145 g. Medians were compared by Mann-Whitney test with Holm-Bonferroni correction. Log-transformed values of geometric means were compared with one-way ANOVA. Values with different letters are significantly different (*P* < 0.05).

b Half of the LOD (2.5 CFU/fruit) was assumed as the pathogen level for fruits that did not yield L. monocytogenes.

c One of the fruits had one serotype IVb-v1 isolate and one 1/2b isolate.

### Serotyping and PFGE.

Out of the 94 isolates from fruits (one isolate from each of the 56 fruits that yielded L. monocytogenes, and one additional isolate from 38 out of these 56 fruits) subject to serotyping, 76 isolates were serotype IVb-v1 (12 from nectarines and 64 from peaches), and 18 isolates were serotype 1/2b (8 from nectarines and 10 from peaches). Serotype IVb-v1, a 4b variant, is recognized as serotype 4b by standard serotyping using antiserum but differs from other serotype 4b isolates by PCR-based serotyping due to acquisition of a 6.3-kb DNA fragment (13, 21). Out of the 38 fruits that had two serotyped isolates per fruit, 5 fruits contained a mixture of both serotype IVb-v1 and 1/2b isolates. The 4 clinical isolates, identified by PulseNet with isolation dates indicating possible exposure to the contaminated fruits (16), were also serotype IVb-v1. Out of the 17 environmental isolates, 3 isolates were serotype IVb-v1, and 14 isolates were serotype 1/2b.

PFGE analysis of serotype IVb-v1 isolates, including the 4 clinical isolates and a subset of food and environmental isolates, revealed an identical profile (Table 2; see also Fig. S1 in the supplemental material). PFGE analysis of a subset of serotype 1/2b isolates from food and environmental samples identified 8 different PFGE profiles which did not match any clinical isolates in PulseNet, with isolation dates indicating possible exposure to the contaminated fruits (16). One environmental isolate (CFSAN024093) had a PFGE profile different from all other isolates (Table 2) and belonged to serotype 1/2b.

**TABLE 2:** L. monocytogenes isolates from food, environment, and patients

Source	Sample identifier	Serotype	PFGE profile (AscI/ApaI)^*a*^	NCBI accession no. or SRA identifier
Illinois patient	PNUSAL000957	IVb-v1	AscP0/ApaP0	SRR1562157
South Carolina patient	PNUSAL000730	IVb-v1	AscP0/ApaP0	SRR1393979
Massachusetts patient	PNUSAL000870	IVb-v1	AscP0/ApaP0	SRR1534987
Minnesota patient	PNUSAL001024	IVb-v1	AscP0/ApaP0	SRR1597487
Nectarine^*b*^	CFSAN023085	IVb-v1	AscP0/ApaP0	SRR1554259
	CFSAN023086	IVb-v1	AscP0/ApaP0	SRR1554256
Yellow nectarine, organic	CFSAN023490	1/2b	NA	SRR1553725
	CFSAN023476	1/2b	AscP1/ApaP1	SRR1553871
	CFSAN023477	1/2b	AscP2/ApaP2	SRR1553850
	CFSAN023489	IVb-v1	AscP0/ApaP0	SRR1553739
	CFSAN023491	IVb-v1	AscP0/ApaP0	SRR1553882
	CFSAN023506	IVb-v1	AscP0/ApaP0	SRR1553851
	CFSAN023475	IVb-v1	AscP0/ApaP0	SRR1553866
	CFSAN023478	IVb-v1	AscP0/ApaP0	SRR1553750
Yellow nectarine	CFSAN023457	1/2b	AscP2/ApaP2	SRR4090659
White nectarine cv. 1	CFSAN023473	IVb-v1	AscP0/ApaP0	SRR1553779
	CFSAN023472	IVb-v1	AscP0/ApaP0	SRR1553855
White nectarine cv. 2	CFSAN023459	1/2b	AscP1/ApaP6	NZ_CP014252.1
	CFSAN023488	1/2b	NA	SRR1553796
	CFSAN023879	1/2b	AscP1/ApaP6	SRR1574290
	CFSAN023474	IVb-v1	AscP0/ApaP0	SRR1553906
	CFSAN023458	IVb-v1	AscP0/ApaP0	SRR1556285
	CFSAN023460	1/2b	AscP2/ApaP2	SRR1556287
Yellow peach, organic	CFSAN023880	1/2b	AscP1/ApaP6	SRR1574296
	CFSAN023462	IVb-v1	AscP0/ApaP0	SRR1556292
	CFSAN023463	IVb-v1	AscP0/ApaP0	NZ_CP012021.1
	CFSAN023464	IVb-v1	AscP0/ApaP0	SRR1556294
	CFSAN023465	IVb-v1	AscP0/ApaP0	SRR1556293
	CFSAN023466	IVb-v1	AscP0/ApaP0	SRR1556289
White peach	CFSAN023492	IVb-v1	NA	SRR1553856
	CFSAN023505	1/2b	NA	SRR1553784
	CFSAN023484	IVb-v1	AscP0/ApaP0	SRR1553821
	CFSAN023495	IVb-v1	NA	SRR1553791
	CFSAN023496	IVb-v1	NA	SRR1553816
	CFSAN023498	IVb-v1	NA	SRR1553788
	CFSAN023500	IVb-v1	NA	SRR1553907
	CFSAN023483	IVb-v1	AscP0/ApaP0	SRR1553773
	CFSAN023503	IVb-v1	NA	SRR1553792
	CFSAN023507	IVb-v1	NA	SRR1553827
	CFSAN023508	IVb-v1	NA	SRR1553774
	CFSAN023479	IVb-v1	AscP0/ApaP0	SRR1553797
	CFSAN023480	IVb-v1	AscP0/ApaP0	SRR1553820
	CFSAN023481	IVb-v1	AscP0/ApaP0	SRR1553798
	CFSAN023482	IVb-v1	AscP0/ApaP0	SRR1553826
	CFSAN023497	IVb-v1	NA	SRR1553756
	CFSAN023499	IVb-v1	NA	SRR1553804
	CFSAN023501	IVb-v1	NA	SRR1566225
	CFSAN023502	IVb-v1	NA	SRR1553840
	CFSAN023504	IVb-v1	NA	SRR1553867
	CFSAN023493	IVb-v1	NA	SRR1553764
	CFSAN023494	IVb-v1	NA	SRR1553740
White peach organic	CFSAN023469	IVb-v1	AscP0/ApaP0	SRR1556291
	CFSAN023470	IVb-v1	AscP0/ApaP0	SRR1556297
	CFSAN023471	IVb-v1	AscP0/ApaP0	SRR1556295
	CFSAN023882	1/2b	AscP2/ApaP2	SRR1574321
	CFSAN023881	1/2b	AscP2/ApaP2	SRR1574272
	CFSAN023468	IVb-v1	AscP0/ApaP0	SRR1556290
	CFSAN023467	IVb-v1	AscP0/ApaP0	SRR1556296
Environment	CFSAN024089	IVb-v1	AscP0/ApaP0	SRR1571515
	CFSAN024077	IVb-v1	AscP0/ApaP0	SRR1571519
	CFSAN024082	IVb-v1	AscP0/ApaP0	SRR1571539
	CFSAN024090	1/2b	AscP2/ApaP4	SRR1571543
	CFSAN024081	1/2b	AscP2/ApaP2	SRR1571521
	CFSAN024083	1/2b	AscP3/ApaP3	SRR1571546
	CFSAN024087	1/2b	AscP2/ApaP2	SRR1571523
	CFSAN024084	1/2b	AscP1/ApaP1	SRR1571540
	CFSAN024092	1/2b	AscP2/ApaP2	SRR1571544
	CFSAN024091	1/2b	AscP3/ApaP3	SRR1571525
	CFSAN024088	1/2b	AscP4/ApaP3	SRR1571524
	CFSAN024086	1/2b	AscP2/ApaP2	SRR1571522
	CFSAN024079	1/2b	AscP2/ApaP2	SRR1571520
	CFSAN024078	1/2b	AscP2/ApaP4	SRR1571538
	CFSAN024080	1/2b	AscP3/ApaP5	SRR1571514
	CFSAN024085	1/2b	AscP2/ApaP2	SRR1571542
	CFSAN024093	1/2b	AscP5/ApaP7	SRR1571545

a NA, PFGE information not available (i.e., not performed).

b Variety information not available.

### SNP-based analyses.

**FIG 2:**
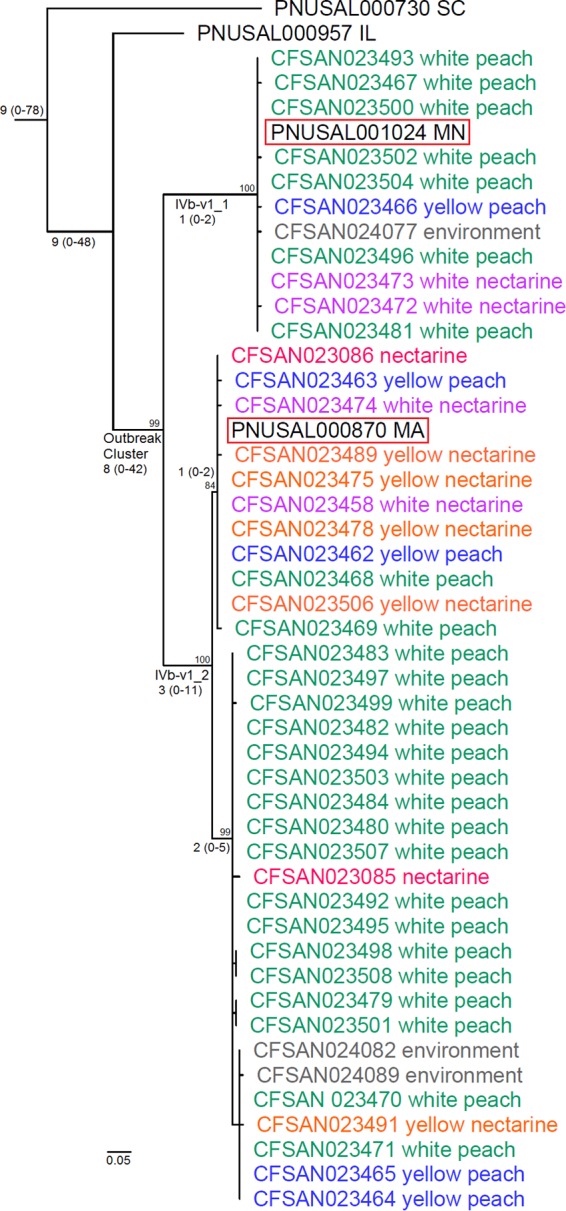
Phylogenetic tree of serotype IVb-v1 isolates constructed from SNPs identified by the CFSAN SNP Pipeline, using CFSAN023463 as the reference. All isolates in the tree were indistinguishable by PFGE. Clinical isolate identification (ID) is followed by the abbreviation of the state where it was isolated. For other isolates, the isolate ID is followed by the food and environmental source of the isolate. Isolates were from patients (black), white peaches (green), yellow peaches (blue), white nectarines (purple), yellow nectarines (orange), nectarines of unidentified variety (red), and environment (gray). The outbreak-associated clinical isolates are highlighted in red boxes. The median, minimum, and maximum pairwise SNP differences among isolates of major clades are shown near the root of each clade, with the minimum and maximum in parentheses. Bootstrap values are shown near major nodes.

The complete genome of CFSAN023463 consists of a single contig of 2,939,733 bp (G+C content, 38%) representing the complete chromosome with no plasmids. The overall coverage was 139×. The outbreak cluster (serotype IVb-v1) consisted of two clades, designated clade IVb-v1_1, containing the Minnesota clinical isolate, and clade IVb-v1_2, containing the Massachusetts clinical isolate (Fig. 2). Each clade contained isolates from fruits of different varieties/lots and their packing environment. The clinical isolates from Illinois and South Carolina, although indistinguishable by PFGE from other serotype IVb-v1 isolates, were placed outside the outbreak cluster (Fig. 2); this was consistent with the epidemiological findings and the wgMLST phylogeny showing that these patients could not be linked to the contaminated stone fruits (16).

The pairwise SNP differences ranged from 0 to 2 (median, 1 difference) among clade IVb-v1_1 isolates, and these ranged from 0 to 11 (median, 3 differences) among clade IVb-v1_2 isolates (Fig. 2). The pairwise SNP differences ranged from 35 to 42 (median, 38 differences) between clade IVb-v1_1 isolates and clade IVb-v1_2 isolates. The pairwise SNP differences ranged from 47 to 48 (median, 47.5 differences) between the Illinois clinical isolate and clade IVb-v1_1 isolates, and these ranged from 40 to 46 differences (median, 43 differences) between the Illinois clinical isolate and clade IVb-v1_2 isolates. The pairwise SNP differences ranged from 70 to 78 between the South Carolina clinical isolate and the stone fruit isolates, indicating that this isolate was more distant from the outbreak cluster (Fig. 2).

In general, it did not appear that the WGS clades or subclades were strongly associated with fruit varieties because isolates from different fruit varieties were distributed across both clades IVb-v1_1 and IVb-v1_2 (Fig. 2). The facility locations of environmental samples were not made available for us to establish any association between WGS clades and different facility locations or to determine whether the contamination was persistent or transient. Twenty-six SNPs (12 nonsynonymous, 13 synonymous, and 1 noncoding) and 56 SNPs (29 nonsynonymous, 24 synonymous, and 3 noncoding) distinguished the outbreak cluster from the Illinois clinical isolate and the South Carolina clinical isolate, respectively (see Table S1 in the supplemental material). Some SNPs were in proteins, including internalins, flagellar hook proteins E and L, and a few surface proteins with yet-unidentified functions (see Table S1). Among all 47 outbreak-associated isolates, we identified 58 polymorphic loci (containing 37 nonsynonymous, 14 synonymous, and 7 noncoding SNPs), some of which were in proteins involved in bacteriocin protection, stress response, and surface anchoring (see Tables S2 and S3 in the supplemental material).

**FIG 3:**
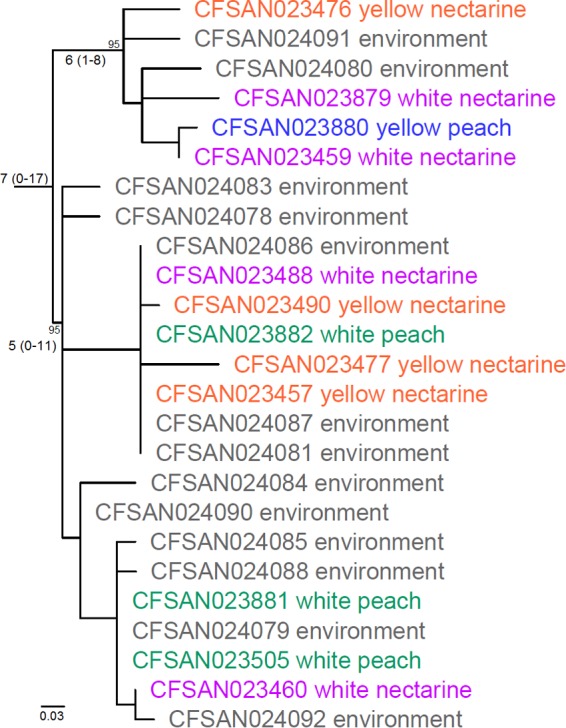
Phylogenetic tree of serotype 1/2b isolates, except CFSAN024093, constructed from SNPs identified by the CFSAN SNP Pipeline, using CFSAN023459 as the reference. The isolate ID is followed by the food and environmental source of the isolate. Isolates were from white peaches (green), yellow peaches (blue), white nectarines (purple), yellow nectarines (orange) and environment (gray). The median, minimum, and maximum pairwise SNP differences among isolates of major clades are shown near the root of each clade, with the minimum and maximum in parentheses. Bootstrap values are shown near major nodes.

The complete genome of CFSAN023459 consists of three replicons: a circular chromosome of 3,039,887 bp (G+C content, 38.1%), a 12,949-bp plasmid (G+C content, 36.4%), and a 52,687-bp plasmid (G+C content, 35.1%). The overall coverage was 119×. The MLST-matched outgroup (CFSAN010068, as described below) and serotype 1/2b isolates, excluding CFSAN024093, differed by at least 250 SNPs in the chromosome, indicating a relatively distant relationship (see Fig. S2 in the supplemental material). We therefore removed CFSAN010068 and analyzed only the stone fruit 1/2b isolates, except CFSAN024093, for the precise determination of SNPs (Fig. 3). The pairwise SNP differences in the chromosome among the serotype 1/2b isolates except CFSAN024093 ranged from 0 to 17 differences (median, 7 differences), indicating a very close relationship among all isolates, despite possessing 7 PFGE profiles. There were 40 polymorphic loci in the chromosome among serotype 1/2b isolates (see Table S4 in the supplemental material), which contained 24 nonsynonymous, 10 synonymous, and 6 noncoding SNPs (see Table S5 in the supplemental material). CFSAN024093 was genetically very distant from other serotype 1/2b isolates during preliminary analysis (data not shown) and thus was not included in the analyses illustrated in Fig. 2 and 3.

### Prophage, clonal group, *inlA*, and methylation analyses.

One incomplete putative prophage of CFSAN023463 was identified by PHAST (36) (Table 3). BLAST analysis of this putative prophage against all serotype IVb-v1 draft genomes, including those from the two epidemiologically unrelated clinical isolates, had >99% query coverage and 100% sequence identity. This was also consistent with the SNP analyses showing that none of the SNPs among serotype IVb-v1 isolates were in the putative prophages (see Tables S1 to S3 in the supplemental material). Four putative prophages of CFSAN023459 were identified by PHAST (36) (Table 3). BLAST analysis of these 4 putative prophages against serotype 1/2b draft genomes, except CFSAN024093, yielded >95% coverage and >99% sequence identity. The variations were mostly due to small insertions/deletions that could be caused by incomplete coverage of prophages by draft sequencing. This is also consistent with the WGS analyses showing that only one of the SNPs among serotype 1/2b isolates except CFSAN024093 was in the putative prophages (see Tables S4 and S5 in the supplemental material). In contrast, BLAST analysis of CFSAN023459 prophages 1, 2, 3, and 4 against CFSAN024093 yielded 100%, 3%, 0%, and 35% coverage, respectively, indicating significant prophage divergence between CFSAN024093 and other stone fruit-associated serotype 1/2b isolates. BLAST analysis of CFSAN023459 prophages 1, 2, 3, and 4 against the MLST-matched outgroup (CFSAN010068, GenBank accession no. NZ_CP014250.1) yielded 100%, 5%, 0%, and 43% coverage, respectively, indicating significant divergence between CFSAN010068 and stone fruit-associated serotype 1/2b isolates.

**TABLE 3:** Putative prophages in the complete genomes of CFSAN023463 and CFSAN023459 identified by PHAST (36)

Strain	Prophage ID	Length (kbp)	Prophage completeness	Positions^*a*^	Most common match, accession no.
CFSAN023463	1	22.9	Incomplete	134411–157386	Listeria A118, NC_003216
CFSAN023459	1	22.9	Incomplete	118524–141499	Listeria A118
	2	32.4	Intact	689648–722048	Listeria A118
	3	27.6	Incomplete	1799035–1826710	Lactobacillus iA2, NC_028830
	4	57.7	Intact	2439092–2496851	Listeria vB_LmoS_293, NC_028929

a Position based on each complete genome.

*In silico* MLST analysis showed that all serotype IVb-v1 isolates had ST382, and all but one serotype 1/2b isolates had ST5, part of clonal complex 5 (CC5). ST382 is a singleton, not a CC, because no other isolates that differ from ST382 by one allele have been observed in the Pasteur MLST database (http://bigsdb.pasteur.fr/listeria/listeria.html) as of August 2016. ST382 was alternatively designated epidemic clone IX (13), and CC5 was alternatively designated epidemic clone VI (13, 37). One serotype 1/2b isolate, CFSAN024093, belonged to singleton ST392. The *inlA* sequences in the serotypes IVb-v1 and 1/2b isolates did not have premature stop codons. The methyltransferases in both CFSAN023463 and CFSAN023459 used the *N*_6_-methyladenine (m6A) methylation (see Table S7 in the supplemental material).

## DISCUSSION

### This incident represented the first reported outbreak and recall associated with fresh whole stone fruits due to the contamination of L. monocytogenes.

Fresh whole fruits have now been confirmed to be transmission vehicles for L. monocytogenes, with outbreaks and recalls linked to contaminated cantaloupes (38), apples (3), mangoes (39), sliced apples (40), and melons (41). Prior to this outbreak, the risk of L. monocytogenes contamination in stone fruit had not been extensively studied, probably because stone fruits have characteristics that do not support the growth of L. monocytogenes. Peach and nectarine pulp generally have a pH below 4.0 (42), which is not favorable for Listeria growth. Collignon and Korsten (43) showed that L. monocytogenes artificially inoculated onto peach surfaces at initial levels of 3 and 5 log CFU/fruit rapidly decreased during the first 6 days of refrigerated storage, although they also established that the L. monocytogenes levels did not significantly drop from the 6th day to the 20th day (final day) of storage at refrigerated temperatures.

In this study, the levels of L. monocytogenes in 99% of the fruits (104/105) were <500 CFU/fruit. Given that the average rinsing recovery rate was 75% to 85% (our unpublished data), the actual levels of L. monocytogenes on the fruits were likely <700 CFU/fruit. The remaining fruit contained L. monocytogenes of 2,850 CFU/fruit, and thus the actual level was likely <4,000 CFU/fruit. Due to the relatively long incubation period of listeriosis and relatively short shelf-life of many ready-to-eat foods, it is difficult to obtain samples from the same lots as the food consumed by the case-patients. Thus, we collected fruits from different lots that were produced in the implicated packing facility and determined the prevalence and levels of L. monocytogenes. It is important to note that the fruits analyzed in our study were not fully ripe and would have needed extra days to reach a stage of ripeness for human consumption. These fruits had also not been distributed to commerce, so it is possible that the behavior of L. monocytogenes on these fruits might be different from that on fruits released to commerce. These variables need to be taken into account when using this data set for future risk assessment. Further, the diversity among L. monocytogenes subpopulations suggests that the observation in this study may only apply to subpopulations of L. monocytogenes most similar to those involved in this outbreak, not necessarily to the entire species. In this outbreak, the Massachusetts patient was between 80 and 89 years old, and the Minnesota patient was between 70 and 79 years old (metadata provided in the GenomeTrakr database under the BioSample ID). A recent study on the 2010 to 2015 U.S. multistate ice cream outbreak showed that the levels of L. monocytogenes were ≤20, ≤50, and ≤100 most probable number (MPN)/g in 92.3%, 98.4%, and 99.8% of the ice cream, respectively (44, 55). That study showed that the geometric means of L. monocytogenes in 7 contaminated lots of ice cream ranged from 0.15 to 7.1 MPN/g. These ice cream samples were linked to a cluster of 4 elderly patients hospitalized with underlying conditions prior to exposure to the contaminated ice cream (44, 55). Contaminated meat or cheese linked to a 2012-2013 Austria outbreak contained L. monocytogenes at <30 CFU/g (45). Contaminated cheese linked to a 2012 U.S. multistate outbreak contained L. monocytogenes at ∼9.0 × 10^3^ to ∼3.75 × 10^6^ CFU/g (median, 4.77 × 10^4^ CFU/g) (4). Contaminated foie gras from 3 unopened samples linked to a 2012-2013 Spain outbreak contained L. monocytogenes at 5.2 × 10^4^ CFU/g (56). These data could help us gain better understanding of the risk associated with L. monocytogenes contamination.

The use of ALOA and RAPID'L. mono agar was critical in the enumeration. We investigated esculin-based agars, e.g., polymyxin-acriflavin-lithium chloride-ceftazidime-aesculin-mannitol (PALCAM) and modified Oxford agar, but the background flora on the fruits made the plate counting difficult. In contrast, ALOA and RAPID'L. mono agar allowed easier differentiation between L. monocytogenes and background flora. We observed presumptive Listeria spp. that were not L. monocytogenes. A Listeria innocua isolate was also recovered from the company A environment (data not shown), confirming the value of using L. monocytogenes-specific chromogenic agars.

### WGS helped linking clinical isolates to the recalled fruits and separated them from PFGE-matched, but epidemiologically unrelated, isolates.

Following the positive pathogen findings in fruits, initial epidemiological investigation, and PFGE analyses, WGS was performed in real time to help identify clinical cases linked to the contaminated fruits (14, 16). Our study illustrates how using SNP-based WGS analyses enabled us to exclude two epidemiologically unrelated clinical cases that were indistinguishable by PFGE from outbreak-associated isolates. The CFSAN SNP Pipeline is a reference-based SNP identification tool. Technically, a reference genome could be one of the isolates associated with the outbreak or an existing genome in the GenomeTrakr database that is genetically close to the isolates being analyzed; in any case, using a complete genome could maximize the resolution power. In the present study, we chose complete genomes of the stone fruit isolates as the references.

The WGS analyses described in the present study and that in the epidemiological report (16) were based on completely different phylogenetic algorithms. They generated identical phylogeny for the same set of isolates, further increasing the confidence in the WGS analyses. The nationwide screening of illnesses based on onset dates and PFGE matches did not identify any patients possibly linked to serotype 1/2b isolates from the stone fruits (16). This could be partially attributed to the lower incidence of serotype 1/2b (18 isolates) than serotype IVb-v1 (76 isolates) in stone fruits with our sampling scheme, especially in peaches (10 serotype 1/2b isolates and 64 serotype IVb-v1 isolates), which had higher levels of L. monocytogenes than those in nectarines. Nonetheless, we still could not exclude the possibility that serotype 1/2b isolates caused unreported illnesses, especially considering that isolates from the same clonal group have been repeatedly involved in previous listeriosis outbreaks as discussed below. With only two colonies picked from each of the 38 fruits, we identified 5 fruits containing both serotypes IVb-v1 and 1/2b; thus, it is possible that more than 5 fruits were contaminated by both serotypes. Therefore, we could not exclude the possibility that the case-patients were coinfected with both serotypes, but that only serotype IVb-v1 was isolated, partially due to its higher incidence. The environmental isolates collected from company A clustered with the food and clinical isolates, indicating that the packing facility was contaminated; however, available environmental samples were only from the packing facility, and specific sampling locations were not available; thus, we cannot determine whether fruit contamination was due to bacteria persistent in the facility or transient contamination originating from sources outside the facility, e.g., fruit orchards.

In most previous listeriosis outbreaks, clusters of illnesses were recognized as an outbreak, and patients were interviewed before the food or environmental source was confirmed by microbial source tracking. In contrast, the investigation and recognition of this outbreak started from the positive pathogen findings in foods (14, 16). One limitation with such an approach is that the identification of clinical cases can be affected by the sampling bias of the food products. We sampled 105 fruits from 7 lots and also obtained fruit and environmental isolates collected by company A; however, we did not have any evidence to determine whether there were L. monocytogenes strains of other genotypes in other lots of the fruits.

WGS data in the present study also provided valuable information about the genetic diversity among isolates involved in a common-source outbreak or isolates associated with foods manufactured in a single packing facility. Previous studies have identified such diversity to be 0 to 5 SNPs (6, 46), 5 to 10 SNPs (47, 48), 10 to 20 SNPs (7), and 20 to 30 SNPs (9), although we need to keep in mind that different studies might target slightly different regions of the genome and employ different bioinformatics tools or parameters to identify SNPs. The Illinois clinical isolate and the outbreak-associated isolates differed by 40 to 48 SNPs, similar to the number of SNP differences (up to 42) among outbreak-associated isolates. However, the WGS phylogeny clearly placed the Illinois clinical isolate outside the outbreak cluster. Similarly, the number of wgMLST allelic differences (up to 47) between the Illinois clinical isolate and the outbreak cluster was similar to the number of allelic differences (up to 43) among isolates in the outbreak cluster, but the Illinois clinical isolate was clearly placed outside the outbreak cluster by wgMLST (16). This indicates that the SNP threshold or the genetic diversity of isolates should not serve as the purpose of outbreak case definition; rather, it should always be combined with WGS phylogeny and epidemiologic evidence to identify strain relationships.

The SNP differences (≤48 SNPs) between outbreak-associated isolates and PFGE-matched, but epidemiologically unrelated, isolates were smaller than those between the stone fruit-associated serotype 1/2b isolates and MLST-matched outgroup (>250 SNPs) and those between the outbreak-associated isolates and MLST-matched, but epidemiologically unrelated, isolates in several previous studies (6, 7). This is consistent with the observation that PFGE possessed more discriminatory power than MLST (37). Therefore, the inclusion of PFGE-matched and epidemiologically unrelated isolates demonstrated the high resolution of WGS and also allowed the identification of SNPs highly specific to the outbreak isolates; many of the SNPs were in surface proteins (see Tables S1, S3, and S5 in the supplemental material), possibly associated with virulence (49).

### Putative prophages were conserved among isolates from the fruits and their packing environment.

Our WGS analyses revealed one putative prophage in the complete genome of CFSAN023463 and four putative prophages in the complete genome of CFSAN023459. The PHAST-identified prophage 4 included a 43.1-kbp *comK* prophage (between positions 2453301 and 2496449), indicating that the PHAST results need to be manually examined before any future in-depth analysis. These putative prophages showed a heavily mosaic nature, because each region contained proteins that were similar to multiple different Listeria phages and phages from other species (see Table S6 in the supplemental material). We used fully closed reference genomes to maximize the resolution power in identifying prophage variations. The >95% query coverage of BLAST analyses between the reference genomes and all but one draft genome indicated that our draft sequencing provided good coverage for prophage regions. BLAST analyses showed that all isolates except CFSAN024093 contained these putative prophages, and the SNP-based WGS analyses revealed 0 and 1 SNP in the putative prophages among serotype IVb-v1 isolates and 1/2b isolates, respectively (see Tables S1 to S5 in the supplemental material). Our finding is consistent with previous observations suggesting that prophages could serve as markers for epidemiology of L. monocytogenes. Specifically, for MLST-matched isolates, prophages were conserved among those involved in a single outbreak/incident or those resident in a facility for a relatively short period of time, but they were more diverse among those involved in different outbreaks/incidents or persistent in a facility for a relatively long period of time (7, 48, 50, 51). In this study, there were no major variations in the putative prophages between the outbreak-associated isolates and the two PFGE-matched, but epidemiologically unrelated, isolates, consistent with some previous findings suggesting that prophage variations and PFGE possessed similar discriminatory power (50, 51). The prophages of all but one stone fruit-associated serotype 1/2b isolate were conserved, but they differed significantly from the MLST-matched outgroup CFSAN010068 from a different outbreak, confirming previous observations that prophages among MLST-matched isolates from different incidents could have significant divergence (50, 51). More whole-genome sequences, especially complete genomes, are needed in order to better understand the Listeria prophage variations among isolates in the same outbreak or those from the same facility.

### WGS data provided information on clonal groups of the isolates from fruits and environment.

The serotype IVb-v1 isolates in this study belonged to singleton ST382, which was not observed in two large-scale surveys studying over 8,000 L. monocytogenes isolates from multiple sources and geographic locations (52, 53). However, ST382 had been associated with a 2014-2015 U.S. multistate caramel apple outbreak and a 2015-2016 U.S. multistate packaged leafy green salad outbreak (13). The singleton ST382 thus represents an emerging clonal group of L. monocytogenes. All but one serotype 1/2b isolate in this study were MLST ST5, part of CC5. The same ST had been involved in a 2011 U.S. multistate cantaloupe outbreak (37, 38), a 2013 U.S. Hispanic-style cheese outbreak (13, 32), a 2010 to 2015 U.S. multistate ice cream outbreak (1, 13), a 2012-2013 Austria outbreak linked to meat and/or cheese (45) (*in silico* MLST performed in our study using published WGS but not described in Materials and Methods), and a 1996 Canada imitation crabmeat outbreak (37, 54). Thus, CC5 appears to be a widely distributed clone contaminating a variety of food products and processing environments and causing outbreaks.

### Conclusions.

The SNP-based WGS analysis provided further discrimination between outbreak-associated isolates and epidemiologically unrelated clinical isolates that were indistinguishable by PFGE. The study highlights the importance to combine WGS with epidemiological evidence to for identifying outbreak-associated isolates. The enumeration data could be useful for future risk-based characterization of L. monocytogenes.

## Supplementary Material

Supplemental material
