# Supplementary material for: Listeria monocytogenes in Stone Fruits Linked to a Multistate Outbreak: Enumeration of Cells and Whole-Genome Sequencing
Source: Appl Environ Microbiol. 2016 Nov 21;82(24):7030–40. doi: 10.1128/AEM.01486-16 (PMC5118914; doi:10.1128/AEM.01486-16)
Supplement: Supplemental material [file supp_82_24_7030__index.html]

Supplemental material 

# Listeria monocytogenes in Stone Fruits Linked to a Multistate Outbreak: Enumeration of Cells and Whole-Genome Sequencing

## Supplemental material

- Supplemental file 1 -

  Pulsed-field gel electrophoresis gel images of isolates from stone fruits and their packing environment analyzed in this study (Fig. S1); phylogenetic tree of serotype 1/2b isolates constructed from SNPs identified by the CFSAN SNP Pipeline using CFSAN023459 as the reference and CFSAN010068 as the outgroup (Fig. S2).

  PDF, 210K
- Supplemental file 2 -

  SNPs specific to the entire set of outbreak isolates when compared to epidemiologically unrelated clinical isolates, their corresponding amino acid changes, genes containing the SNPs, and their encoded proteins (Table S1).

  XLSX, 18K
- Supplemental file 3 -

  SNP matrix of outbreak-associated isolates (Table S2).

  XLS, 73K
- Supplemental file 4 -

  SNPs, amino acid changes, and genes containing the SNPs and their encoded proteins among outbreak-associated isolates (Table S3).

  XLSX, 16K
- Supplemental file 5 -

  SNP matrix of serotype 1/2b isolates except CFSAN024093 (Table S4).

  XLS, 47K
- Supplemental file 6 -

  SNPs, amino acid changes, and genes containing the SNPs and their ecoded proteins among serotype 1/2b isolates except CFSAN024093 (Table S5).

  XLSX, 15K
- Supplemental file 7 -

  Coding sequences of each prophage region identified by PHAST (Table S6).

  XLSX, 24K
- Supplemental file 8 -

  Putative methylation patterns of the two complete genomes identified by PacBio SMRT sequencing (Table S7).

  XLSX, 11K
