# Supplementary material for: Listeria monocytogenes in Stone Fruits Linked to a Multistate Outbreak: Enumeration of Cells and Whole-Genome Sequencing
Source: Appl Environ Microbiol. 2016 Nov 21;82(24):7030–40. doi: 10.1128/AEM.01486-16 (PMC5118914; doi:10.1128/AEM.01486-16)
Supplement: Supplemental material [file AEM.01486-16_zam999117554so1.pdf]

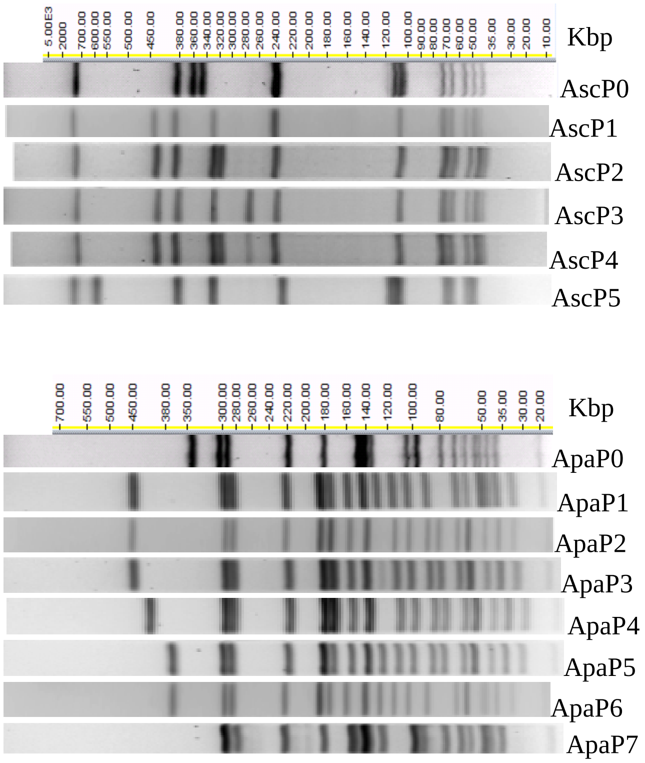

FIG S1 Pulsed-field gel electrophoresis gel images of isolates from stone fruits and their packing environment analyzed in this study.

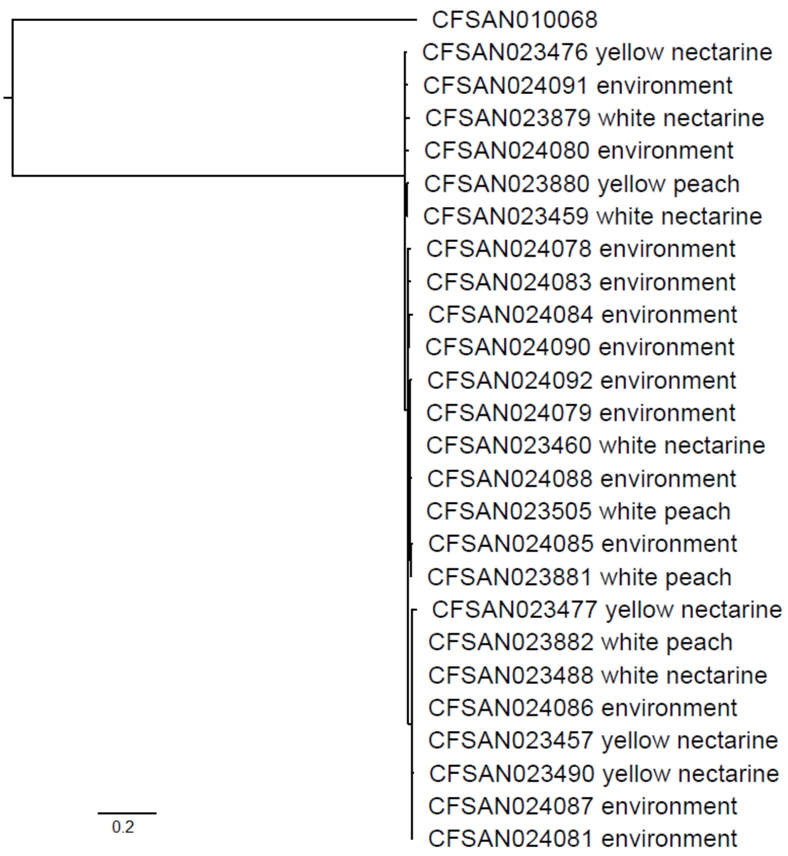

FIG S2 Phylogenetic tree of serotype 1/2b isolates constructed from SNPs identified by the CFSAN SNP Pipeline using CFSAN023459 as the reference and CFSAN010068 as the outgroup. The isolate ID is followed by the food and environmental source of each isolate.
